# Evaluation of the surgical and prosthetic success of All-on-4 restorations: a retrospective cohort study of provisional vs. definitive immediate restorations

**DOI:** 10.1186/s40729-021-00330-1

**Published:** 2021-05-31

**Authors:** Michael Korsch, Winfried Walther, Matthias Hannig, Andreas Bartols

**Affiliations:** 1 Dental Academy for Continuing Professional Development, Lorenzstrasse 7, 76135 Karlsruhe, Germany; 2 Clinic of Operative Dentistry, Periodontology and Preventive Dentistry, University Hospital, Saarland University, Building 73, 66421 Homburg, Germany; 3 Center for Implantology and Oral Surgery, 69120 Heidelberg, Germany; 4 Clinic for Conservative Dentistry and Periodontology, School for Dental Medicine, Christian-Albrechts-University, Kiel, Germany

**Keywords:** All-on-4, Definite, Fixed denture, Implant, Provisional, Restoration

## Abstract

**Background:**

All-on-4 concept allows an immediate restoration, which is frequently a provisional restoration (PR), and will be replaced by a definitive restoration (DR) a few months later. However, this approach involves much higher treatment efforts and costs, compared to a DR immediately after implantation. PRs were mostly incorporated in the introductory phase of the All-on-4 concept in our respective clinics. Today, PRs are only used for referred patients and bimaxillary restorations. The aim of the study was to investigate whether PRs and DRs have comparable success rates.

**Methods:**

A total of 126 patients with 136 All-on-4 restorations supported by 544 implants were included in this retrospective cohort study. The observation period was 1 year. In 42 cases, a PR was placed initially and replaced by a DR 3 months later. In 94 cases, a DR was placed immediately. Biological, technical, and severe (loss of an implant or PR/DR) complications associated with PRs and DRs were compared. The absence of a serious complication was considered a success.

**Results:**

A total of 27 patients were affected by 33 complications, 19 biological (2 PR and 17 DR) and 14 technical (6 in PR and 8 in DR) in the first 3 months. Eight patients had ten severe complications (1 PR and 9 DR). Severe complications were all implant losses. Implant survival rate was 98.2% (99.4% PR and 97.6 DR), and restoration survival rate was 94.4% (97.6% PR and 92.6% DR). Six out of the ten implant losses occurred in the posterior maxillae of male patients. After 3 months, ten complications occurred in six patients within 1 year. One of these complications was an implant loss in the posterior maxillae of a male patient.

**Conclusion:**

PRs and DRs showed comparable complication rates during the observation period. Only in male patients did implant losses occur more frequently in the posterior maxilla.

**Supplementary Information:**

The online version contains supplementary material available at 10.1186/s40729-021-00330-1.

## Introduction

The conventional restoration of the edentulous jaw is a full denture [1], the function and retention of which is limited because it only adheres to the mucosa and no other retention elements are present [2]. Anterior biting or mastication of solid food is usually only possible to a limited extent compared to the natural dentition. By covering sensory and phonetic relevant areas of the oral mucosa, the perception of taste and vocalization can be impaired. This is in contrast to implant-supported restorations, which can be either removable or fixed.

Fixed implant-supported restorations are most similar to the natural dentition since there is no movement of the denture and there is no need to cover the alveolar ridge to support the saddles of restorations. This avoids a limitation of phonetics and sensory perception.

According to the recommendation of the 2014 consensus conference on implantology, eight implants in the maxilla and six implants in the mandible are required as the gold standard for fixed implant-supported restorations [3]. But in many cases, there is an inadequate implant site. In these situations, augmentation procedures must often be performed, sometimes even using autogenous bone from extraoral donor regions such as the iliac crest [4]. This additional treatment considerably increases the surgical risk, postoperative discomfort, and financial burden on the patient.

In 1998, Maló used for the first time the fixed restoration on a reduced number of implants according to the All-on-4 concept. Usually, four implants are inserted to support the fixed restoration. The angulated insertion of the distal implants allows bypassing anatomical structures associated with operative risks, such as the maxillary sinus in the maxilla and the inferior alveolar nerve in the mandible. In this way, alveolar ridge augmentations or sinus floor elevations can be often avoided and the prosthetic support zone can be extended into the posterior region [5]. The reduced number of implants and the technical design of the restoration lead to comparatively moderate costs for the patient and are roughly equivalent to those of a bar restoration. A further advantage is an immediate restoration and loading, which is possible because of the immediate splinting of the implants, which significantly reduces the treatment time compared to conventional restorations. Additionally, it appears that the implant survival rate for fixed restorations (89–100% after 5 years) in the edentulous jaw is higher than for removable restoration (24.9–100% after 5 years) [6].

In the traditional loading protocol, implants must be allowed to heal after placement until sufficient osseointegration has taken place. Therefore, in many cases, functional loading by a dental prosthesis is often applied after osseointegration of the implants is completed. For the period between implant placement and osseointegration, patients are usually provided with a temporary removable restoration, which is functionally inferior to a fixed restoration. Due to the primary splinting of the immediate restoration in the All-on-4 concept, a fixed denture is possible despite the initial lack of osseointegration. Peñarrocha et al. showed that there is no significant difference in survival rates and marginal bone resorption between immediate and delayed restorations in edentulous jaws with fixed, full-arch restorations [7].

Since there is a risk of insufficient or no osseointegration after implant placement, the All-on-4 concept in most cases includes an immediate temporary fixed restoration [8, 9]. After successful osseointegration, the definitive fixed restoration is fabricated [9]. In the event of implant loss during the healing phase, the additional costs of an immediate definitive fixed restoration would be much higher, since the required repeated implant placement might also require the replacement of the complete definitive restoration. In addition to the uncertainty of successful osseointegration after immediate loading, the earlier absence of computer-aided design/computer-aided manufacturing (CAD/CAM) fabrication of frameworks resulted in immediate restorations being avoided or impossible. However, if osseointegration is successful, the additional treatment burdens and costs with an immediate provisional restoration would be higher than those of an immediate restoration with a definitive denture. To the knowledge of the authors, there are currently no comparative studies available in which All-on-4 restorations have been restored immediately after implantation, either with provisional fixed dental restorations or with the definitive fixed restoration.

In our working group, provisional fixed restorations were mostly incorporated in the introductory phase of the All-on-4 concept in our respective clinics. However, the concept was subsequently further developed so that a definitive fixed restoration with a CAD/CAM-milled definitive cobalt-chromium-molybdenum framework could be incorporated just within 24 h after implantation. The use of provisional fixed restorations has now become significantly less common. Today, provisional restorations are only used in two situations in our institutes. On the one hand, with referred patients, the definitive restoration is incorporated by the referred family dentist, on the other hand, with bimaxillary restorations. For logistical reasons, a definitive restoration after 1 day is only possible in one jaw with bimaxillary restorations. The other jaw is provisionally restored and only final after osseointegration of the implants.

The aim of this retrospective study was, therefore, to compare provisional and definitive immediate restorations within the healing period of 3 months after implantation according to the All-on-4 concept with regard to biological and prosthetic complications and thus to investigate whether the immediate fixed definitive restoration with dental prostheses is reasonable as part of the All-on-4 treatment protocol.

## Materials and methods

For the present retrospective study, the electronic medical records of patients from the Academy for Continuing Professional Development in Karlsruhe and the Centre for Implantology and Oral Surgery in Heidelberg were evaluated. The study included consecutively all adult patients who received either a provisional or a definitive All-on-4 immediate restoration with Nobel Biocare implants (Nobel Biocare, Kloten, Switzerland) in the period from June 1, 2014, to September 30, 2019. The observation period was 1 year. All surgical procedures were performed by the same surgeon.

This resulted in two observation groups:

- Group 1 (PR): provisional immediate restoration with a metal wire-reinforced acrylic resin-based restoration
- Group 2 (DR): immediate restoration with definitive restoration with CAD/CAM-milled metal framework

Inclusion criteria:

- Patients with threatened or existing edentulism
- Patient who advocated an All-on-4 restoration and declined alternative therapies
- Observation period of 1 year
- Patients who were treated in the Academy for Continuing Professional Development in Karlsruhe or the Centre for Implantology and Oral Surgery in Heidelberg
- Patient who received either a provisional or a definitive All-on-4 immediate restoration with Nobel Biocare implants in the period from June 1, 2014, to September 30, 2019
- Only patients who were treated by the same surgeon

Exclusion criteria:

- Patients who did not have an observation period of 1 year after implantation
- Patients in whom already existing osseointegrated implants were integrated into new All-on-4 construction
- Patients who were not treated in the Academy for Continuing Professional Development in Karlsruhe or the Centre for Implantology and Oral Surgery in Heidelberg
- Patients with severe systemic diseases, tumors and cancer, untreated and/or poorly controlled diabetes mellitus, and severe cardiovascular diseases; patients undergoing immunosuppressive drug therapies affecting bone metabolism; and patients after irradiation of the head and neck region
- Patients with more than four implants per restoration
- Patients who were treated by another surgeon

The study was conducted in conformity with the Declaration of Helsinki and the Professional Code for Physicians of the Medical Council of the State of Baden-Württemberg. The Ethics Committee of the Baden-Württemberg Medical Council reviewed the study and approved it (Ref. Ident. No. F-2020-056-z; date of decision 30/06/2020).

### Surgical and prosthetic treatment

To assess the bone height and width of the alveolar ridge, a cone-beam computed tomography was performed preoperatively in all cases. In brief, a preoperative wax-up was prepared for all treatment cases, based on which drilling templates for the surgical procedure were produced to ensure the best possible prosthetic alignment of the implants. All surgical procedures were performed by the same surgeon.

In cases of threatened edentulousness, the implants were inserted simultaneously with the extraction of the teeth. In all cases, the alveolar ridge was fully exposed with a crestal incision. All implants were placed using the drilling template. Only “Nobel Biocare Active” implants were used in the maxilla and “Nobel Biocare Replace Conical Connection” implants (Nobel Biocare, Kloten, Switzerland) in the mandible. The implant diameters used were 3.5 mm and 4.3 mm. To achieve primary stability of the implants, the implants were placed with a torque of 35 N/cm. Then, depending on the inclination of the implants, angled (17° or 35°) or straight multi-unit abutments (Nobel Biocare) were placed. The wound was then closed with non-resorbable suture material (Supramid® 5/0, Serag Wiessner) and an open impression (Impregum soft®, 3M, Saint Paul, USA, and Miratray® Implant, Duisburg, Germany) of the implants including bite registration taken directly afterward. The provisional or definitive immediate restoration was placed within 24 h after surgery. In cases of a bimaxillary restoration, a maximum of one restoration was placed as the definitive immediate restoration. This was due to the maximum possible capacity of one milled CAD/CAM framework per 24 h.

### Design of the All-on-4 constructions

**Fig. 1:**
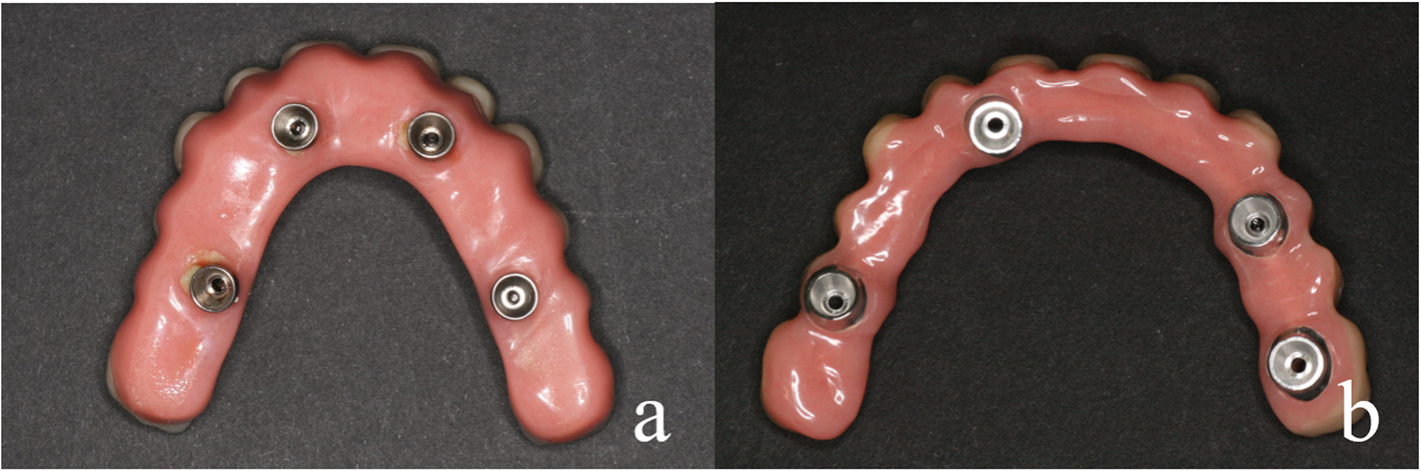
**a** A basal view of a provisional fixed immediate All-on-4 restoration. The base is very wide because the stability of the denture depends on the resin and the hand-bent stainless steel wire inside the restoration. **b** A basal view of a definitive fixed immediate restoration. The design of this restoration can be much more slender since its stability depends on a CAD/CAM-milled stainless steel framework. The teeth are fixed to this framework by polymerization

Provisional fixed All-on-4 restorations consisted of prefabricated composite teeth (Phonares II, Ivoclar Vivadent GmbH, Ellwangen, Germany) which were fixed in an acrylic resin base (PalaXpress, Kulzer GmbH, Hanau, Germany). For stability reasons, the base was designed larger than that of the definitive All-on-4 restoration. A 2-mm-thick hand-bent steel wire was additionally inserted inside the restoration to minimize the risk of constructional fractures (Fig. 1a). In definitive All-on-4 designs, immediate final stability was achieved by a CAD/CAM-milled (NT-TRADING GMBH & CO. KG, Karlsruhe, Germany) cobalt-chrome-molybdenum (CoCrMo6 Fräsrohling/ZENOTEC NP, WIELAND Edelmetalle GmbH, Pforzheim, Germany) framework. As with the provisional restoration, prefabricated composite teeth were fixed to an acrylic resin base (Fig. 1b). The CAD/CAM framework made it possible to design the base of the final restorations much more delicate right from the start. After 3 months, provisional All-on-4 restorations were replaced by the definitive restoration, which was designed in the same way as the immediate definitive restoration, provided there was sufficient osseointegration at that time. During incorporation, the screw canals of the restorations were provisionally closed for the 3-month healing phase with foam pellets or Teflon tape to secure the screw heads and occlusally with Telio onlay (Ivoclar Vivadent, Schaan, Liechtenstein).

**Fig. 2:**
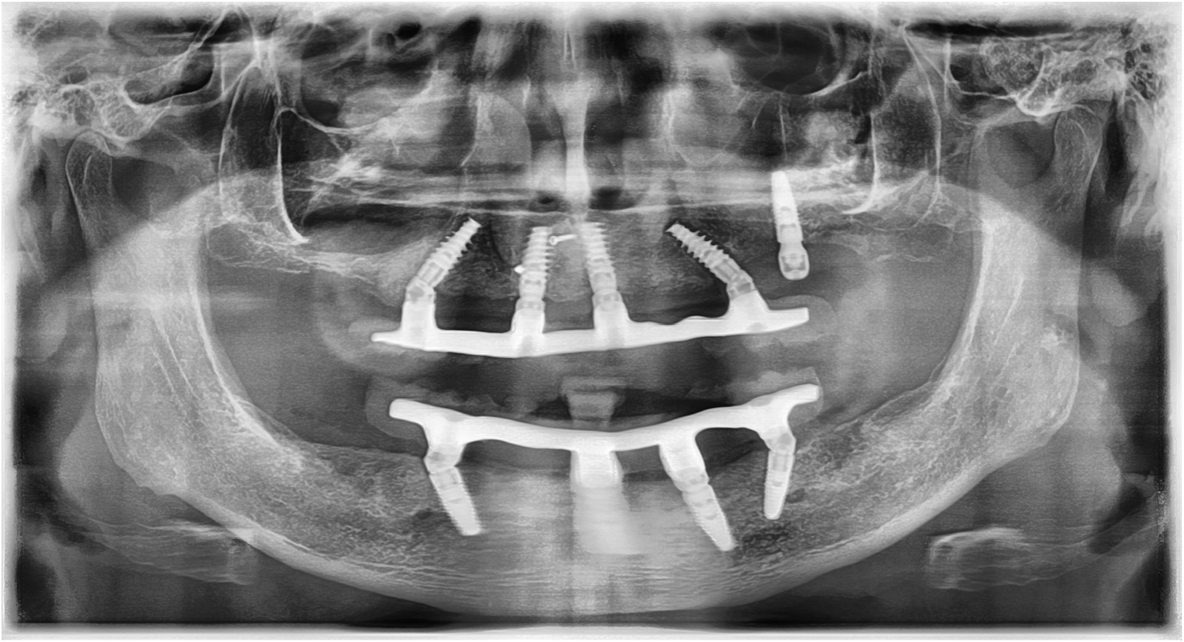
The procedure of what was done in case of an implant loss. The X-ray shows an All-on-4 restoration on four implants. The angled implant in the 2nd quadrant was loosened. For this reason, another implant with an external sinus elevation was placed in this area. During the entire healing period of this implant, the All-on-4 restoration was fixed on the remaining four implants, so that there was no load on the newly inserted implant

**Fig. 3:**
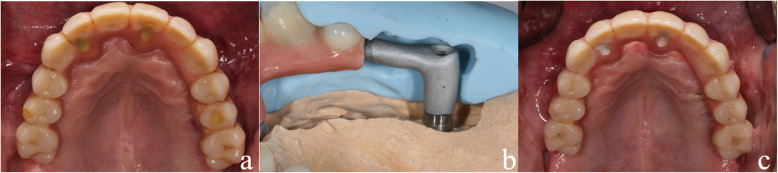
**a** The restoration of the case in Fig. 2 before explantation. During the entire healing time of the dorsal implant in the 2nd quadrant, the restoration was fixed on the three osseointegrated implants and the one loosened implant in the 2nd quadrant. **b** After successful osseointegration of the dorsal implant in the 2nd quadrant, the dentures were adapted to the new situation. For this purpose, the dorsal element of the restoration in the 2nd quadrant, which was the connection to the loosened implant, was separated. A connection piece was cast on the implant that was subsequently inserted and welded to the rest of the restoration. **c** The situation after incorporation of the adapted restoration

**Fig. 4:**
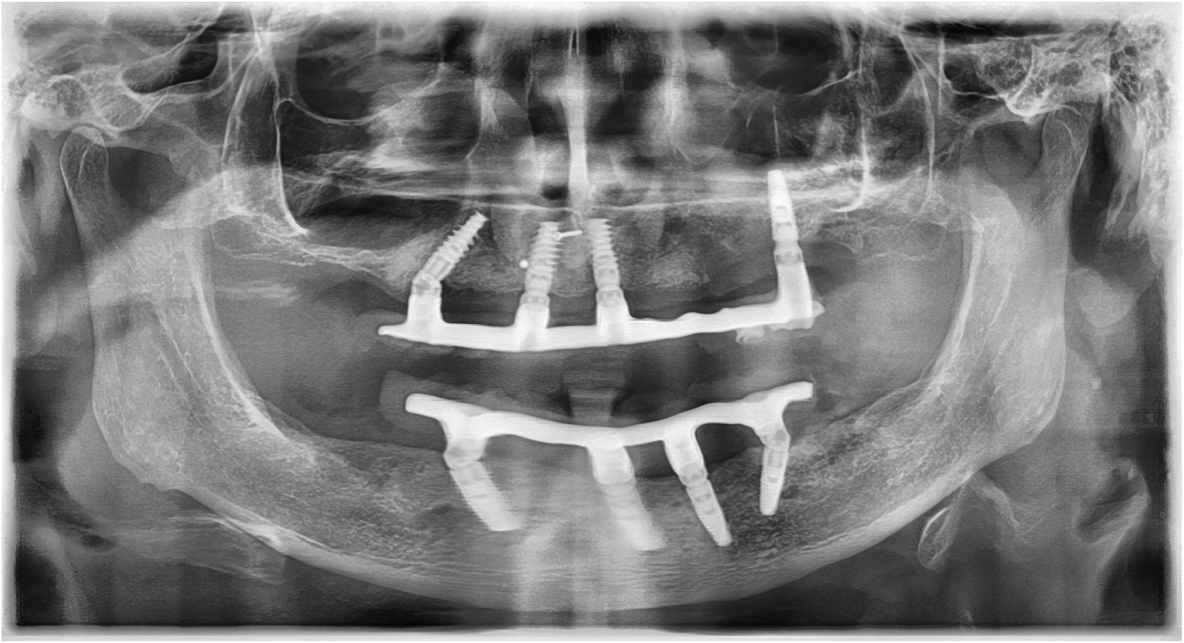
This X-ray records the integration of the redesigned restoration of the case in Figs. 2 and 3. The loosened implant was explanted

In cases in which implants were lost during the healing period, an attempt was always made to replace these implants with reimplantation. For reimplantations in the upper molar area, a simultaneous external sinus lift had to be performed in most cases. In these cases, a Nobel Biocare Replace implant was always used, as this is slightly conical in the area of the implant shoulder (Fig. 2). Nobel active implants are tapered in the area of the implant shoulder and pose the risk of insufficient primary stability or even of slipping into the maxillary sinus if the bone height is low. In cases of provisional acrylate-based immediate restorations, these were repaired with acrylate and reintegrated. In the case of a definitive immediate restoration, the CAD/CAM-milled framework was separated and then welded to a newly milled framework section using laser welding technology (Figs. 3 and 4).

### Follow-up

The patients were closely monitored. All patients were followed up after 1, 2, and 3 weeks. After 3 months, the dentures were screwed down and the implants checked. Complications were found at these times. In principle, patients who had noticed complications could present themselves at any time. After 1 year, all patients were given a routine check-up as standard. Complications could thus be found even after 1 year.

#### 3-month follow-up inspection

In all patients, the restoration was screwed down after 3 months. As part of this, the peri-implant tissue of all implants was probed at four locations (mesial, distal, oral, and buccal) with a periodontal probe. Only those implants were rated as successful if no probing depth was more than 2 mm. Implant movement was ruled out with dental tweezers. These implants were considered a failure. A panoramic X-ray was then taken to assess the osseointegration of the implants and possible bone loss. Implants with sufficient osseointegration were rated as successful. Finally, the restoration was screwed back on.

#### Observed outcome parameters

In all treatment cases, biological and technical complications occurred in the first year after implantation was observed. This corresponded to the planned healing interval after which, in the case of the provisional restoration, the definitive restoration is scheduled with undisturbed healing of the implants according to our treatment protocol. After 3 months, both observation groups were generally subjected to an implant check-up, during which the restoration was removed to assess the osseointegration of the implants.

### Severe complications

Severe complications had an impact on the survival rate of implants or restorations. We have defined a complication that was led during the follow-up to the loss of an implant or a restoration as a severe complication.

### Non-severe complications

Non-severe complications had no impact at all during the follow-up on the survival rate of implants and restorations and resulted in little or no additional effort (maximum 30 min).

### Survival rate of implants or restorations

A therapy was rated as successful if no severe complication occurred. Since non-severe complications, as described above, did not affect the results of an implant or a restoration, we did not consider them as a failure. The survival rate was defined as the percentage of implants or restorations which fulfill the success criteria.

### Biological complications

Biological complications included all complications affecting the hard tissue (bone) and the soft tissue (mucosa).

These can cause both severe and non-severe complications. The biological complications included postoperative complications such as postoperative bleeding, infections with and without suppuration, implant loss, wound dehiscence, increased peri-implant bone loss, and hypesthesia of the nervus alveolaris inferior for mandibular restorations. Implant loss was considered a severe biological complication, potentially resulting in total loss of the restoration or significant adjustments to the restoration in the context of the All-on-4 concept.

### Technical complications

Technical complications included complications related to the implant, abutment, implant/abutment interface, or the All-on-4 restoration (framework and composite teeth). In detail were counted loosening of the abutment or restoration, fracture of prosthetic screws, implant fractures, chipping of the prefabricated composite teeth, fracture of the All-on-4 restoration, and loss of the resin cover of the screw channels.

### Data evaluation

Data were compiled in Excel and analyzed with IBM SPSS Statistics 21 (SPSS Inc., Chicago, IL, USA, on Windows XP). The following data from the electronic medical record were excerpted for the study documentation: age and sex, previous diseases of the patient, systemic diseases, data of the investigated implant treatment, the subsequent prosthetic therapy, and data of the maintenance therapy, furthermore, any possible complications. The statistical methods included cross-tabulations with chi-squared for categorical data. Mean values were compared by *t*-tests and ANOVA. All evaluations were computed on patient, restoration, and/or implant level. A probability of error of *p* < 0.05 was interpreted as significant. During the evaluation, a distinction was made between the groups, maxilla and mandibular, anterior and posterior implants, and between sexes.

## Results

A total of 126 patients with 136 All-on-4 restorations and 544 implants were included in this study. The data sets for all patients were complete. This did not lead to a dropout. All non-severe, severe, biological, and technical complications of all cases could be evaluated during the 3-month healing period after implantation and after 1 year. In group 1 (PR), 34 patients with 42 restorations and 168 implants were identified, and in group 2 (DR), 92 patients with 94 restorations and 376 implants were identified (Table 1). No statistically significant difference was found between the groups in terms of gender distribution. The patient’s age ranged between 33 and 90 years. The patients of group 1 were younger than the patients of group 2 (63.4 vs. 68.3 years). Eighty-six restorations were placed in the maxilla and 50 in the mandible (Table 1). Of the 34 patients with a provisional restoration, 17 were referred; in 13 patients, a provisional restoration was incorporated in the introductory phase of the All-on-4 concept in our institutes, and four patients who were not referred received bimaxillary treatment. In two of the bimaxillary treated patients, the upper jaw and, in the other two, the lower jaw received immediate restoration. The opposing jaw was provisionally restored immediately and finally after 3 months.

**Table 1:** Baseline characteristics of the study groups at the time of restoration

	Total	Study group	
		Group 1 (PR)	Group 2 (DR)
**Patients** ***n*** **(%)**	**126 (100)**	34 (27)	92 (73)
**Gender (male)** ***n*** **(%)**	**58 of 126 (46.0)**	15 of 34 (44.1)	43 of 92 (46.6)
**Mean age at the time of surgery (*years*)**			
**Mean (SD)**	**67.2 (10.7)**	62.4 (12.8)	69.0 (9.7)
**Restoration** ***n*** **(%)**	**136 (100)**	42 (30.9)	94 (69.1)
**Implants** ***n*** **(%)**	**544 (100)**	168 (30.9)	376 (69.1)
**Restoration maxilla** ***n*** **(%)**	**86 of 136 (63.2)**	27 of 42 (64.3)	59 of 94 (62.8)

*PR* provisional restoration, *DR* definite restoration

### Complications during the 3-month healing period after implantation

A total of 33 complications (Table 2) were observed for both groups in 27 restorations of 27 patients (Table 3). There were no significant differences in the complication rate between groups 1 and 2 at implant, restoration, or patient level (Table 1). Immediate and late implantations did not affect the results.

**Table 2:** Individual biological and technical complications per group within the healing period of 3 months

	Total	Study group	
		Group 1 (PR)	Group 2 (DR)
**Biological complications** ***n***	**19**	2	17
Dehiscence *n*	**1**	1	0
Temporary hypesthesia *n*	**2**	0	2
Infection *n*	**4**	0	4
Abscess (*n*)	**(3)**	(0)	(3)
Fistula (*n*)	**(1)**	(0)	(1)
Sharp bone edge *n*	**1**	0	1
Secondary bleeding *n*	**1**	0	1
Implant loss *n*	**10**	1	9
**Technical complications** ***n***	**14**	6	8
Chipping/fracture of composite teeth *n*	**11**	5	6
Fracture prosthetic screw *n*	**1**	0	1
Abutment loosening *n*	**1**	1	0
Loss of the cover SC *n*	**1**	0	1

*PR* provisional restoration, *DR* definite restoration, *Loss of the cover SC* loss of the resin cover of the screw channel

**Table 3:** Detailed patient-related complication rates of the study groups

	Total	Study group		Sign.
		Group 1 (PR)	Group 2 (DR)	*p*-value
**Total complications**				
**Patient level** ***n*** **(%)**	**27 of 126 (21.4)**	6 of 34 (17.6)	21 of 92 (22.8)	*χ*^2^ = 0.395, *p* = 0.529
**Restoration level** ***n*** **(%)**	**27 of 136 (19.9)**	6 of 42 (14.3)	21 of 94 (22.3)	*χ*^2^ = 1.184, *p* = 0.277
**Implant level** ***n*** **(%)**	**33 of 544 (6.1)**	8 of 168 (4.7)	25 of 376 (6.6)	*χ*^2^ = 0.726, *p* = 0.394
**Biological complications**				
**Patient level** ***n*** **(%)**	**17 of 126 (13.5)**	2 of 34 (5.9)	15 of 92 (16.3)	*χ*^2^ = 2.310, *p* = 0.129
**Restoration level** ***n*** **(%)**	**17 of 136 (12.5)**	2 of 42 (4.8)	15 of 94 (16.0)	*χ*^2^ = 3.327, *p* = 0.068
**Implant level** ***n*** **(%)**	**19 of 544 (3.5)**	2 of 168 (1.2)	17 of 376 (4.5)	*χ*^2^ = 3.822, *p* = 0.051
**Technical complications**				
**Patient level** ***n*** **(%)**	**11 of 126 (8.7)**	4 of 34 (11.8)	7 of 92 (7.6)	*χ*^2^ = 0.538, *p* = 0.463
**Restoration level** ***n*** **(%)**	**11 of 136 (8.1)**	4 of 42 (9.5)	7 of 94 (7.4)	*χ*^2^ = 0.168, *p* = 0.681
**Implant level** ***n*** **(%)**	**14 of 544 (2.6)**	6 of 168 (3.6)	8 of 376 (2.1)	*χ*^2^ = 0.965, *p* = 0.326
**Non-severe complications**				
**Patient level** ***n*** **(%)**	**20 of 126 (15.9)**	5 of 34 (14.7)	15 of 92 (16.3)	*χ*^2^ = 0.048, *p* = 0.827
**Restoration level** ***n*** **(%)**	**20 of 136 (14.7)**	5 of 42 (11.9)	15 of 94 (16.0)	*χ*^2^ = 0.380, *p* = 0.538
**Implant level** ***n*** **(%)**	**23 of 544 (4.2)**	7 of 168 (4.2)	16 of 376 (4.2)	*χ*^2^ = 0.002, *p* = 0.962
**Severe complications**				
**Patient level** ***n*** **(%)**	**8 of 126 (6.3)**	1 of 34 (2.9)	7 of 92 (7.6)	*χ*^2^ = 0.910, *p* = 0.340
**Restoration level** ***n*** **(%)**	**8 of 136 (5.9)**	1 of 42 (2.4)	7 of 94 (7.4)	*χ*^2^ = 1.346, *p* = 0.246
**Implant level** ***n*** **(%)**	**10 of 544 (1.8)**	1 of 168 (0.6)	9 of 376 (2.4)	*χ*^2^ = 2.081, *p* = 0.149

*PR* provisional restoration, *DR* definite restoration, *p-values indicate a statistically significant difference with *p* < 0.05

Of the 33 complications, 19 were biological complications (Table 2). These were distributed among 17 restorations in 17 patients. There were no significant differences at implant, restoration, or patient level.

Fourteen of the 33 complications were technical complications in both groups (Table 2). There were no significant differences between groups 1 and 2 at implant, restoration, or patient level (Table 3). All these technical complications could be repaired without total loss of the restoration.

There were no significant differences between groups 1 and 2 at implant, restoration, or patient level in terms of non-severe complications (Table 3).

In both groups, a total of ten severe complications occurred in eight All-on-4 restorations and eight patients. In all cases, it was implant losses, each of which required reimplantation and subsequent adaptation and repair of the restoration. There were no significant differences between observation groups 1 and 2 at implant, restoration, or patient level (Table 3). The implant losses were always loosened implants without sufficient osseointegration. Implant survival rate (implant level) was 98.2% (99.4% PR and 97.6 DR), and restoration survival rate (restoration level) was 94.4% (97.6% PR and 92.6% DR).

In all cases, additional implants could be placed to replace the loosened implants (Fig. 2). Until complete osseointegration of the newly inserted implants, the existing restoration could be left on the remaining implants in all cases. Following the osseointegration of the newly placed implants, the definitive restoration was fabricated and incorporated on the four fully osseointegrated implants in group 1. The loosened implants were removed on the day the new restoration was placed. In group 2, after osseointegration of the newly placed implants, the restoration was repaired as described in the “Materials and methods” section and thus retained (Figs. 3 and 4).

A comparison between All-on-4 restorations in the maxilla and mandible, in which provisional and definitive restorations were evaluated together, showed no significant differences at the implant and restoration levels, neither in the total number of complications nor the biological, technical, or non-severe and severe complications.

A comparison of implants in the anterior and posterior region regardless of whether the restorations were temporary or definitive All-on-4 restorations showed no significant differences in the total number of complications, technical and non-severe complications. However, posterior implants were significantly more frequently affected by biological (*χ*^2^ = 4.417, *p* = 0.036) and severe (*χ*^2^ = 6.520, *p* = 0.011) complications than anterior implants. The comparison of anterior and posterior implants separated by maxilla and mandible showed, though, that posterior implants in the maxilla were significantly more frequently affected by complications, especially biological (*χ*^2^ = 3, df 9.922, *p* = 0.019) and severe (*χ*^2^ = 3, df 11.763, *p* = 0.008) complications than all anterior implants or the posterior implants in the mandible. Eight out of ten implant losses affected posterior implants in the maxilla. For the total number of complications, technical and non-severe complications, there were no significant differences in the localization of the restoration or the implants.

A comparison between the sexes in terms of complication rates showed at the patient, restoration, and implant levels, there were no significant differences in biological, technical, and non-severe complications. The total number of complications was only significant on the patient level (*χ*^2^ = 3.965, *p* = 0.046). However, serious complications were significantly more frequent in male patients (eight of ten implant losses) at the patient (*χ*^2^ = 5.913, *p* = 0.015), restoration (*χ*^2^ = 5.168, *p* = 0.023), and implant levels (*χ*^2^ = 5.177, *p* = 0.044). Six of the total ten implant losses occurred in six male patients on posterior implants in the maxilla.

### Complications within the first year after the healing period of 3 months

After the healing period of 3 months, all implants were provided with a definitive restoration. From this point in time to the 1-year follow-up, ten complications occurred in six patients (Table 4). One of these complications was an implant loss (former PD). All other complications could be resolved with reasonable effort. Composite teeth where chipping occurred were smoothed or composite was applied. Fractured prosthetic screws have been replaced.

**Table 4:** Individual complications per group within the first year after the healing period of 3 months

	Study group		Region	
	Group 1 (former PR)	Group 2 (former DR)		
**Patient 1**	X		26	Loss of implant
**Patient 2**	X		36	Chipping
**Patient 3**	X		36	Fracture prosthetic screw
			46	Fracture prosthetic screw
**Patient 4**	X		22	Chipping
			26	Chipping
**Patient 5**	X		13 15	Fracture prosthetic screw
			15	Fracture prosthetic screw
			23	Fracture prosthetic screw
**Patient 6**		X	16	Abutment loosening

*PR* provisional restoration, *DR* definite restoration

### Survival rate of implants and restorations within 1 year

Serious complications with 11 implant losses occurred in nine patients within 1 year. The overall survival rate on implant level was 98.0% (PR 98.8% and DR 97.6%) and on the restoration level 93.3%.

Forty-three (13 PR and 30 DR) restorations in the maxilla were placed in women. One of these DRs suffered from a severe complication with two-implant losses. The survival rate in the maxilla in women was 97.7% (100% PR and 96.7% DR). Twenty-seven restorations (7 PR and 20 DR) were in the mandible in women. No serious complications occurred in any of the cases. The survival rate for both PRs and DRs was 100%.

There were also 43 (14 PR and 29 DR) restorations in the maxilla in men. Severe complications with 7 implant losses occurred in 7 restorations (1 PR and 6 DR) within 1 year. The survival rate was 83.7% (92.9% PD and 79.3% DR). Twenty-three restorations (8 PR and 15 DR) were in the maxilla in men. Only in one DR did a severe complication with two-implant losses occurred. The survival rate in men in the mandible was 95.7% (100% PR and 93.3% DR).

## Discussion

In this retrospective study, 136 All-on-4 restorations supported by a total of 544 implants in 126 patients were investigated. The surgical and prosthetic successes of provisional and definitive All-on-4 immediate restorations were compared. Based on the complication rate, PRs and DRs seem equally successful. The survival rate was over 90% in women in the maxilla and mandibular and in men in the mandibular. In men, due to the higher rate of implant loss in the posterior maxilla, the survival rate in the maxilla was lower than 90%. In cases of implant loss, the restoration can be adapted to a new situation. In the literature, we could not find a comparison between provisional and definitive All-on-4 restorations.

Two thirds (*n* = 86, 63.2%) of all constructions were placed in the maxilla. Out of the 136 restorations placed immediately after implantation, 42 (30.8%) were provisional. The number of complications was very different between the two groups in some sub-areas, but not significantly. The different group sizes had a not inconsiderable influence on this. While there were 168 implants in group 1, there were 376 implants in group 2, i.e., more than twice as many.

A total of 33 complications (6.1% at the implant level) for both groups occurred during the healing period (interval between implantation insertion and 3-month follow-up). Out of these complications, 19 (3.59%) were biological and 14 (2.6%) technical. Ten (1.8%) of the 19 biological complications were severe complications. These severe complications were exclusively cases of implant loss. In the literature, implant losses are also cited as the most common biological complication [8]. The non-severe biological complications were four infections, one secondary bleeding, one dehiscence, two temporary hypesthesias, and once a sharp bone edge.

Eleven of the 14 technical complications were chipping or rather fractures of the composite teeth. Publications on the subject indicate that restoration and composite tooth fractures are the most common technical complications, followed by screw loosening [10, 11]. There were no significant differences in total, biological, technical, non-severe, or severe complications between the two groups at the patient, restoration, and implant levels. No significant differences were found at the patient and restoration levels. The type of restoration (PR vs. DR) had only a marginal influence on the complication rates. Implant loss was considered a severe complication in this study. However, implant loss does not necessarily result in denture loss and remake. Maló found implant survival rates of 93% or 91.7%, respectively, in follow-up periods of 10 to 18 years. The cumulative denture survival rate was 98.8%, though [12].

The implant survival rate is a key success parameter in implant therapy. In the follow-up period of 1 year, the implant survival rates were 98.0% for all cases, 98.8% for provisional immediate restorations, and 97.6% for definitive immediate restorations. The values are comparable to those of conventional implantations without immediate restorations [13]. For the All-on-4 concept, there are several studies documenting implant loss, denture survival, and marginal bone resorption for periods of up to 7 years. Retrospective studies of All-on-4 restorations conducted by Maló et al. showed cumulative implant survival rates of 95.4% at 7 years [14]. For removable implant-supported dentures, Romeo et al. found a cumulative survival rate of 95.7% at 7 years [15]. These data, based on extended observation periods, confirm that All-on-4 restorations can be as successful as conventional restoration concepts in terms of implant survival.

The comparison of anterior and posterior implants did not show any significant differences in total and technical and non-severe complications. Strikingly, however, posterior implants showed significantly more biological and severe complications. Eight severe complications occurred in posterior regions within the healing period of 3 months and nine within a year and only two in anterior regions over the entire observation period. The severe complications also had a critical influence on the total number of biological complications. In the literature, straight and angled implant insertion methods do not differ in implant loss rates or peri-implant bone loss [16].

Another division into four regions (anterior vs. posterior implants in maxillae vs. mandibles) showed significantly more total, biological, and severe complications in posterior maxillae. Eight out of ten cases of implant loss occurred in posterior maxillae within the healing period. The technical complications did not differ significantly. The survival rates of straight and angled implant insertion methods are equal, according to the literature [14, 17]. However, no distinction is made between maxillary and mandibular angled implants.

Gender did not significantly influence total, biological, and technical complications. But male patients were significantly more often affected by severe complications at the patient, restoration, and implant levels. In the literature, there are indications that complication rates are higher in male patients than in female patients [12].

Six out of the ten cases of implant loss occurred in the posterior maxillae of six male patients. The occlusal force in the molar regions is up to 30% higher in men than in women [18]. So the higher implant loss rates in posterior maxillae of male patients may be caused by a combination of higher occlusal stress, lower bone density in posterior maxillae [19], and angulation of posterior implants. Generally, bruxism may influence implant survival rates [20].

The main factor determining the success rate of restorations was the survival rate of the implants. The survival rate within a year of restorations in the maxilla in women was 97.7% (100% PR and 96.7% DR) and 100% for both PRs and DRs in the mandible. The survival rate in men in the mandible was 95.7% (100% PR and 93.3% DR) and 83.7% (92.9% PD and 79.3% DR) in the maxilla. When implants were lost in our study, we were able to adapt primary definitive All-on-4 restorations to the new situation using newly inserted implants. A complete loss of the restoration, which would have led to high costs, was avoided in this way.

A limitation of the study is the short observation period of 1 year. After the 3-month follow-up, all patients including those with temporary restorations had a definitive restoration. Most complications occurred in the healing period of 3 months. After this period, there were relatively few complications. A longer observation period would not have had any scientific added value in terms of the comparison between PRs and DRs in this study.

## Conclusion

The type of restoration (PR vs. DR) had only a marginal influence on total, biological, technical, non-severe, and severe complications. Implant loss occurred mainly in the posterior maxillae of male patients.

## Supplementary Information

**Additional file 1:.** Raw data

## Data Availability

All data generated or analyzed during this study are included in this published article [and its supplementary information files].

## Abbreviations

ANOVA: Analysis of variance
CAD/CAM: Computer-aided design/computer-aided manufacturing
DR: Definitive restoration
PR: Provisional restoration
